# Association between low bone mass and the serum RANKL and OPG in patients with nephrolithiasis

**DOI:** 10.1186/s12882-018-0960-z

**Published:** 2018-07-11

**Authors:** Asieh Mansour, Maryam Aboeerad, Mostafa Qorbani, Amir Pejman Hashemi Taheri, Mohamad Pajouhi, Abbas Ali Keshtkar, Bagher Larijani, Mohammad Reza Mohajeri-Tehrani, Mohammad Reza Ganji

**Affiliations:** 1 Endocrinology and Metabolism Research Center, Endocrinology and Metabolism Clinical Sciences Institute, Tehran University of Medical Sciences, Tehran, Iran; 2 Non-Communicable Diseases Research Center, Alborz University of Medical Sciences, Karaj, Iran; 3 Chronic Diseases Research Center, Endocrinology and Metabolism Population Sciences Institute, Endocrinology and Metabolism Research Institute, Tehran University of Medical Sciences, Tehran, Iran; 4 Radiology department, Shariati hospital, Tehran University of Medical Sciences, Tehran, Iran; 5 Department of Health Science Education Development, School of Public Health, Tehran University of Medical Sciences, Tehran, Iran; 6 Nephrology Research Center, Shariati Hospital, Tehran University of Medical Sciences, Tehran, Iran

**Keywords:** Low bone mass, Osteopenia, Osteoporosis, BMD, OPG, RANKL, Nephrolithiasis

## Abstract

**Background:**

Nephrolithiasis is a risk factor for Osteopenia and osteoporosis. Receptor activator of nuclear factor kappaB ligand (RANKL) and osteoprotegerin (OPG) regulate bone remodeling and osteoclastogenesis. This study aimed to evaluate the relation between serum OPG, RANKL concentration, and bone mineral density (BMD) in patients with kidney stone disease.

**Methods:**

Forty-four nephrolithiasis patients with either low bone mass or normal BMD (considered control group) were enrolled in this study. BMD was measured at lumbar spine (L1-L4) and femoral neck by dual-energy X-ray absorptiometry (DEXA). The serum OPG and RANKL were determined using the ELISA method.

**Results:**

The median levels of serum OPG were significantly higher in nephrolithiasis patients with low bone mass compared to the nephrolithiasis patients with normal BMD (3.9 pmol/l versus 3.1 pmol/l; *P* = 0.03), respectively. Negative correlation was detected between bone densities of femoral neck and OPG in patients with nephrolithiasis (*r* = −.0344, *P* = 0.02).

**Conclusion:**

The present study showed that high serum fasting OPG levels may be indicative of femoral neck BMD in patients with nephrolithiasis.

## Background

A number of studies have provided evidence for the association between kidney stone and lower BMD. In men, stronger associations have been reported between the history of nephrolitasis and low BMD [1, 2]. In addition, population- based studies have shown an increased fracture risk among stone formers [2, 3]. In fact, several in vitro and rodent studies have revealed that proinflammatory cytokines such as interleukin-6 (IL-6), interleukin-1 (IL-1), and tumor necrosis factor-alpha (TNF-α) are involved in the pathogenesis of osteoporosis. These cytokines increase the synthesis of RANKL and macrophage colony-stimulating factor (M-CSF) [4]. In the mid-1990s, it was discovered that OPG/RANKL/RANK system plays a main role in the regulation of bone remodeling [5]. OPG is expressed on the osteoblasts and osteocytes, which binds to RANK and stimulate the activity and differentiation of osteoclasts [6]. Due to the soluble receptor role of OPG for RANKL, it can prevent RANKL from interacting with RANK through binding to RANKL [5, 7], suggesting that it plays an antiresorptive role [8].

The aim of this study was to define the role of RANKL and OPG in the development of nephrolithiasis- related reduced bone mineral density. So we compared nephrolithiasis patients with low bone mass to nephrolithiasis patients with normal BMD to identify risk factors for the development of low bone mass in such patients.

## Methods

### Study subjects

Potential participants attending the specialty clinic at Shariati university hospital, located in the city of Tehran-Iran, were invited to participate in this study. Eligible subjects with nephrolithiasis who had given written informed consent were entered into the study. All subjects had to be over the age of 18, willing and able to give written consent to the study, and had a diagnosis of nephrolithiasis. Participants were excluded if they had hyperparathyroidism, thyroid gland dysfunction, renal tubular acidosis, malignancy, longstanding corticosteroid intake. Other reasons for exclusion were use of estrogen, progesterone, bisphosphonate, calcitonin, heparins, and anti- inflammatory drugs, calcium, vitamin D and vitamin C supplements and treatment with thiazides diuretics. Information regarding nephrolithiasis in preceding years and medication use was retrieved by interviewing patients. The duration of kidney stone was considered as the difference between the current age of the subject and the age at the time of the first diagnosis of kidney stone. Ethics Committee approval was gained from Tehran University of Medical Sciences (code: EC- 00308) and all participants provided written informed consent.

### Measurement of BMD

BMD measurement of the lumbar spine (L1-L4) and one femur was performed by dual – energy X- ray absorptiometry (DEXA) using Lunar DPX-MD device (Lunar Corporation, Madison, Wisconsin, 53,713. USA). Results were expressed as absolute values (g/cm2), Z-score and T-score. The threshold for establishing a diagnosis of Low bone mass was based on the World Health Organization definition.

### Blood tests

Samples were drawn from subjects after overnight fasting of 10–12 h at the baseline of the study. Serum fasting and plasma EDTA tubes were immediately centrifuged for 15 min at 2500×g. Serum and plasma specimens were frozen and stored at − 80 °C until assay. Serum concentrations of creatinine, BUN (Blood Urea Nitrogen), uricacid, calcium, phosphor levels were determined by a colorimetric method using Pars Azmoon kits (Pars azmoon.co, Tehran, Iran). Serum concentration of PTH was measured by Biomenca. Serum levels of vitamin D were assayed by IDS. A 24-h urine sample was taken from subjects in order to evaluate calcium, phosphor, uric acid, citrate, createnine, and urine volume. Urine levels were measured by Pars Azmoon kits (Pars azmoon.co, Tehran, Iran). Serum OPG and RANKL levels were measured by ELISA kit according to the indication of manufacturer (DRG International Inc. (USA)).

### Anthropometric measures

Height (to the nearest 0.5 cm) was measured using wall mounted stadiometer. Weight was taken in minimal clothing and no shoes. BMI was calculated by dividing body weight (in kg)/ height (in m) ^2^. Ultrasonography examinations were performed by an experienced radiologist in Shariati hospital to diagnosis kidney stone.

### Statistical analysis

A *p* value of< 0.05 was considered statistically significant (SPSS Inc. USA). The variables are shown as means ±SD and median (interquartile range). The Kolmogorove Smirnov test of normality was applied. Differences between groups were tested by mann- whitnineys for none -normally distributed variables, unpaired t-test for normally distributed variables. Correlation between variables was assessed by spearman’s rank test. Receiver operator characteristic (ROC) analysis was performed to establish the discriminator performance of OPG in predicting low bone mass in patients with nephrolithiasis.

## Results

Forty four patients with nephrolithiasis (21 men and 23 women) were enrolled in this study. The majority of participants were middle age (mean age 47.5 ± 11.95 years) with a mean 28.94 ± 4.21 (kg/m^2^) BMI. Table 1 displays clinical characteristics between male and female. As shown by table, males had significantly higher median (25th,75th) BUN, creatinine, uric acid, urine creatinine and urine phosphorous (15.8 (14.15,18.45) mg/dl, 1.1(1,1.25) mg/dl, 6.3 (5.65,7.25) mg/dl, 1538 (1150,1789.5) mg/24 h, 832 (663.75,1163.5) mg/24 h, respectively) than in females (14 (11.8,16.3) mg/dl, 0.8 (0.8,0.9) mg/dl, 4.4 (3.3,5.3) mg/dl, 1011 (725,1273.5) mg/24 h, 671 (484.5,814.5) mg/24 h, respectively) (*p* < 0.05).

**Table 1:** Comparison of clinical parameters and lab data between male and female in nephrolithiasis patients

Clinical parameters	Total	Male patients	Female patients	*p*-value
	Median(25th–75th)	Median(25th–75th)	Median(25th–75th)	
Age(years)	47.5 ± 11.94	50.19 ± 12.43	45.04 ± 11.17	0.15
BMI(Kg/m^2^)	28.94 ± 4.21	28.65 ± 4.90	29.2 ± 3.56	0.67
Lab data				
OPG(pmol/l)	3.5(2.9–4)	3.4(2.9–4.3)	3.6(2.8–3.9)	0.61
sRANKL(pmol/l)	0.2(0.1–0.3)	0.2(0.1–0.3)	0.2(0.1–0.3)	0.85
BUN(mg/dl)	15.2(12.98–17.38)	15.8(14.15–18.45)	14(11.8–16.3)	0.02
Creatinie (mg/dl)	0.9(0.8–1.1)	1.1(1–1.25)	0.8(0.8–0.9)	≤ 0.001
Uric acid (mg/dl)	5.45(4.03–6.3)	6.3(5.65–7.25)	4.4(3.3–5.3)	≤ 0.001
Ca(mg/dl)	9.6(8.9–9.98)	9.7(8.95–10.05)	9.5(8.9–9.9)	0.2
P(mg/dl)	3.4(3.13–4.18)	3.3(3–4)	3.6(3.3–4.2)	0.19
Urine volume(ml/24 h)	1350(900–2237.5)	1650(950–2237.5)	1150(825–2250)	0.39
Urine creatinine(mg/24 h)	1227(949.5–1624)	1538(1150–1789.5)	1011(725–1273.5)	0.003
Urine uric acid(mg/24 h)	593(405–804.5)	786(436–865.25)	547(390–760.5)	0.14
Urine calcium (mg/24 h)	209.5(150–327)	193.5(152.5–285)	232(105–340.5)	0.9
Urine phosphorus (mg/24 h)	741.5(592.5–958)	832(663.75–1163.5)	671(484.5–814.5)	0.04
PTH(pg/ml)	32.9(24.65–42.83)	36.1(27.1–41.8)	31.1(20.7–61.4)	0.5
Vitamin D(nmol/l)	36.8(25.45–61.10)	40(27.1–56.7)	31.55(20.5–64.45)	0.41
Urine citrate (mg/24 h)	493(267–740)	427(277–674)	592(225–756.25)	0.3
Na(mEq/l)	137.4(136.8–138.35)	137.3(136.75–138)	137.5(136.8–139)	0.5
eGFR (ml/minute)	102.48(82.27–119.33)	101.98(79.14–117.83)	103.62(82.43–125.85)	0.71
kidney stone disease duration (year)	3.5(1, 11)	3.25(1–8.88)	4.5(1–11.5)	0.66

*eGFR* estimated glomerular filtration rate based on the Creatinine Cockcroft-Gault Equation

Table 2 presents the parameters of BMD, T-score and Z-score in total body, left femur and lumbar spine location and compares these parameters between two genders. The median (25th,75th) BMD of the femoral neck of patients was 0.87 (0.75,0.99) g/cm^2^ whereas the median BMD T-score and BMD Z- score were − 0.6 (− 1.6,0.1) and − 0.7(− 2,-0.2), respectively. There were significant differences in T-score of femur neck and T-score and Z-score of lumbar spine between male and female (*p* < 0.05). As well, higher T-score and Z-score of femur neck and higher T-score and Z-score of lumbar spine were identified in females (− 0.8 (− 1.7, 0.03), − 0.6 (− 1.08,0), − 0.2 (− 1.8,0.25), − 0.35 (0.78,0.1)) than males (− 1.7 (− 2.4,-0.65), − 1.2 (− 1.7,0.35), − 1.7 (− 2.05,-0.75), − 1.6 (− 2.35,0.8)), respectively (Table 2). However, there were no significant differences in values of femur neck, femur total and lumbar spine BMD, and also T- score and Z-score of femur total among two groups.

**Table 2:** Comparison of BMD between male and female in nephrolithiasis patients

Bone mineral density	Total	Male patients	Female patients	*p*-value
	Median(25th,75th)	Median(25th,75th)	Median(25th,75th)	
Femur Neck				
g/cm2	0.87(0.75,0.99)	0.9(0.77,0.99)	0.87(0.75, 0.99)	0.73
T-score	− 1.3 (− 2, − 0.1)	− 1.7(−2.4,-0.65)	− 0.8(− 1.7,0.03)	0.04
Z- score	−.7(−1.5, − 0.2)	− 1.2(− 1.7,-0.35)	− 0.6(− 1.08,0)	0.06
Femur Total				
g/cm2	0.94(0.84,1.03)	0.92(0.83,1.08)	0.95(0.84,1.02)	0.95
T-score	− 0.6(− 1.6, 0.1)	−1.2(− 2,0.1)	− 0.4(− 1.53,0.23)	0.2
Z- score	− 0.4 (− 1.4, 0.2)	− 1.1(− 1.7,0.05)	− 0.3(0.78,0.2)	0.21
Spine				
g/cm2	01.06(0.97,1.18)	1.02(0.96,1.12)	1.15(0.96,1.2)	0.25
T-score	− 1(− 1.9, 0)	1.7(− 2.05,−0.75)	− 0.2(− 1.8,0.25)	0.03
Z- score	−.7(− 2, − 0.2)	− 1.6(− 2.35,0.8)	− 0.35(0.78,0.1)	0.002

There was a significant inverse linear relationship between OPG and femur neck BMDs (*r* = −.0344, *P* = 0.02). OPG didn’t show any significant relation to lumbar spine (*r* = − 0.295, *p* = 0.05) (Table 3). Femoral neck BMDs were negatively correlated with the age of patients (*r* = − 0.544, *p* ≤ 0.001) and kidney stone disease duration (*r* = − 0.41, *p* = 0.002) and positively correlated with the BMI (*r* = 0.341, *p* = 0.02), urine phosphor (*r* = 0.572, *p* = 0.001) and eGFR (*r* = 0.54, *p* ≤ 0.01). Femoral total BMDs were negatively correlated with age (*r* = − 0.369, *p* = 0.009) and positively correlated with BMI (*r* = 0.498, p = 0.001), urine phosphorous (*r* = 0.533, *p* = 0.002) and eGFR (*r* = 0.44, *p* = 0.003). Lumbar spine BMDs were negatively correlated with age (*r* = − 0.338, p = 0.02) and positively correlated with urine phosphor (*r* = 0.437, *p* = 0.01) (Table 3), whereas no other relationship was found between parameters of BMD with BUN, creatinine, uric acid, calcium, phosphor, PTH, sodium, vitamin D, and also urinary biochemical variables such as calcium, uric acid (*p* > 0.05).

**Table 3:** Correlation coefficient of BMD (g/cm2) with clinical characteristics in nephrolithiasis patients

	Femur neck	P-value	Femur total	P-value	Lumbar spine	*P*-value
OPG(pmol/l)	−0.344	0.02	−0.228	0.14	−0.295	0.05
sRANKL(pmol/l)	0.220	0.16	0.122	0.44	0.249	0.1
OPG/RANKL	−0.2	0.19	− 0.16	0.30	− 0.26	0.09
Age(years)	−0.544	00	−0.369	0.009	−0.338	0.02
BMI (Kg/m^2^)	0.341	0.02	0.498	0.001	0.079	0.61
BUN(mg/dl)	−0.257	0.09	−0.205	0.18	−0.126	0.41
Creatinie(mg/dl)	0.05	0.75	0.028	0.86	−0.188	0.22
Uric acid(mg/dl)	−0.09	0.56	−0.159	0.3	−0.231	0.13
Ca(mg/dl)	0.126	0.42	0.072	0.64	−0.011	0.94
Phosphorous (mg/dl)	−0.098	0.53	−0.150	0.33	−0.158	0.31
Urine volume(ml/24 h)	−0.026	0.89	0.001	0.99	0.104	0.57
Urine creatinine (mg/24 h)	0.275	0.13	0.199	0.28	0.110	0.55
Urine uric acid(mg/24 h)	0.308	0.09	0.243	0.18	0.161	0.38
Urine calcium(mg/24 h)	−0.133	0.47	−0.016	0.93	−0.153	0.41
Urine p(mg/24 h)	0.572	0.001	0.533	0.002	0.437	0.01
PTH(pg/ml)	−0.288	0.06	0.215	0.16	−0.227	0.14
Vitamin D(nmol/l)	−0.231	0.15	−0.242	0.13	−0.205	0.2
Na(mEq/l)	−0.218	0.16	−0.133	0.39	−.291	0.05
Urine citrate (mg/24 h)	0.103	0.58	0.105	0.58	0.112	0.55
eGFR (ml/minute)	0.54	≤0.01	0.44	0.003	0.26	0.84
kidney stone disease duration (year)	−.041	0.02	−0.17	0.34	−.024	0.19

Patients were divided into two groups: Group 1 consisted of 23 patients with low bone mass and group 2 included 21 patients with normal BMD (control group). When comparing to control group, those with the decreased BMD were significantly found to have higher OPG levels (3.1(2.8,3.7) pmol/l versus 3.9(2.9,4.4) pmol/l; *P* = 0.03) and lower eGFR (111(100.04,129.6) ml/minute versus 83.96(72.77,112.75) ml/minute; *p* = 0.002) and higher duration of kidney stone disease (1.25(1,10.75) years versus 5.5(3,11) years p = 0.002) (Table 4). Differences in RANKL levels between this two groups didn’t reach statistical significant (*p* = 0.19).

**Table 4:** Comparison of clinical characteristics among nephrolithiasis patients with normal and low bone mass

Clinical characteristics	Normal	Osteoporosis/ Osteopenia	*P*- value
	Median(25th,75th)	Median(25th,75th)	
Age(years)	40(35.5,47)	56(42,64)	0.05
BMI (Kg/m2)	28.95(27.3,30.52)	27.43(24.49,30.39)	0.1
OPG(pmol/l)	3.1(2.8,3.7)	3.9(2.9,4.4)	0.03
sRANKL(pmol/l)	0.2(0.1,0.3)	0.2(0.1,0.2)	0.19
OPG/RANKL	14.5(10.17,29)	20(14,39)	0.08
Na(mEq/l)	137.30(136.65,138)	137.6(136.9139.2)	0.23
BUN(mg/dl)	13.6(12.1,15.9)	16.3(14.1,17.7)	0.25
Creatinie(mg/dl)	0.8(0.8,1.1)	1(0.8,1.3)	0.62
Uric acid (mg/dl)	5.3(4.4,6.1)	5.9(3.9,7)	0.94
Calcium(mg/dl)	9.6(9.1,9.9)	9.4(8.9,10)	0.62
Phosphorous(mg/dl)	3.3(3.25,4.2)	3.5(3.1,4.1)	0.94
Urine volume (ml/24 h)	900(775,1950)	1700(1100,2250)	0.21
Urine creatinine (mg/24 h)	1104(850,1570)	1360(946,1740)	0.59
Urine uric acid (mg/24 h)	702(369,876.5)	572(400,800)	0.52
Urine ca (mg/24 h)	195(99,294)	234(160,352)	0.51
Urine p(mg/24 h)	828(614,1134)	682(590,864)	0.21
PTH(pg/ml)	31.1(19.2,38.6)	37.5(27,61.4)	0.09
Vitamin D(nmol/l)	36.25(20.5,57.83)	36.8(26.15,64.95)	0.54
Urine citrate (mg/24 h)	496.5(259.25,743.75)	356(267,733)	0.67
eGFR(ml/minute)	111(100.04,129.6)	83.96(72.77, 112.75)	0.002
kidney stone disease duration (years)	1.25(1, 10.75)	5.5(3,11)	0.002

There were no significant differences in age and BMI distribution. No significant differences were detected in the levels of sodium, BUN, creatinine, uric acid, calcium, phosphor, PTH, and vitamin D. As well, the differences in urine levels of citrate, phosphor, calcium, uric acid, creatinine, and volume among the two groups were not significant (Table 4).

Using a ROC analysis, our data showed that OPG levels were sensitive for the presence of low bone mass at the femoral neck among individuals with nephrolithiasis. A threshold levels for OPG at 3.38 pmol/l enabled us to detect low bone mass with AUC, sensitivity and specificity of 68% (95%CI: 52–85), 69% (95%CI: 52–82) and 67% (95%CI: 50–80), respectively.

## Discussion

This study showed an increased circulatory OPG in nephrolithiasis patients with reduced femoral neck density. Our data showed that nephrolithiasis patients with reduced BMD have higher serum OPG levels compared to those with normal bone density. No significant association was observed between RANKL and bone densities. Among people with kidney stone, duration of nephrolithiasis is significantly and positively related to the presence of low bone mass especially at femoral neck. In addition, in ROC analysis, OPG levels predicted BMD levels at femur neck in these patients.

To our knowledge, this is the first work that tries to gain some understanding on the role of serum OPG and RANKL in association with the measurements of BMD in nephrolithiasis patients. Nephrolithiasis has been demonstrated to accelerate bone loss; therefore, osteoporosis could be possibly attributed to either the presence of hypercalciuria [9] or decreased dietary calcium intake [10], and elevated serum levels of inflammatory factors such as IL-1, IL-6 and TNF-α [11]. A very recent meta- analysis of 24 case-control studies in patients with nephrolithiasis indicated the lower BMD values at all skeletal sites and in relation to osteoporosis, suggesting a four times increased risk of osteoporosis in those with nephrolithiasis compared to healthy controls [9]. Furthermore, our data has demonstrated a worsen BMD in male patients with nephrolithiasis, as evident by lower T-score at the femur neck and T-score and Z-score at the lumbar spine.

One explanation may be the OPG acts as a decoy substrate to RANKL and RANK competitor; thus, inhibiting the formation and activity of osteoclasts [8, 12]. Some studies have reported the increased plasma OPG levels in patients with nephropathy [13] and osteoporosis [14] compared to those controls. For example, idiopathic hypercalciuria (IH), characterized by the excessive loss of urine calcium, is the most common metabolic abnormality in people in whom kidney stones form [15]. A study by Gomes et al. [16] noted the higher bone expression of both RANKL and OPG in patients with IH than in control subjects. Although the mechanism for the bone effects of OPG is elusive, evidence suggests that OPG may act as a protective factor for osteoporosis [14]. This hypothesis implies that the increased circulating OPG levels in nephrolithiasis patients with reduced BMD may be a compensatory mechanism against other factors that promote bone damages [14], perhaps as a part of response to inflammation in nephrolithiasis.

In addition, we observed that femoral neck BMD was inversely correlated with serum OPG and not the vertebral spine in patients with kidney stones. Bone minerals can be divided into two types: cortical and trabecular. The femoral neck region contains both trabecular and cortical tissue whereas the vertebrae region contains high amounts of trabecular tissue [17–19]. In this study, patients with low bone mass were older, had a higher PTH and had decreased levels of GFR than normal patients.

It is well known that cortical bone turnover is increased in presence of high PTH levels [20]. It is also well known that reduced GFR levels are associated with significant cortical bone loss [21, 22]. In addition the results of a study investigating changes in the bone density of trabecular and cortical bone revealed variable rates of change during aging. Large decreases in trabecular BMD in both genders were reported before age 50, but studies have found a small decrease in bone density at cortical bone before age 50 in men and women [23]. Thus, taken together, we speculate that decreased GFR, elevated PTH levels and higher age in low BMD groups may contribute to the differences in the underlying mechanism relating to OPG levels and BMD of the femoral neck seen in these populations.

There are some limitations to our study. Although this study supported an inverse correlation between circulatory levels of OPG with BMD measurement, which highlights the potential link between nephrolithiasis and reduced bone mass, but due to the cross-sectional nature of our study cautious the causal assumption between them; whether reduced bone mass brings high levels of OPG in nephrolithiasis or that high levels of OPG in nephrolithiasis brings low bone mass. Future longitudinal studies in patients will help to determine whether the OPG provides additive predictive value for femoral neck BMD decline. In addition, A power analysis for OPG levels based on the differences demonstrated in our study between patients with low bone mass (3.14 ± 0.72) and normal BMD (3.68 ± 0.98), with an α of 0.05, resulted in a power of 58%. Thus, it is possible that our relatively small sample size limited our ability to detect true differences and an additional study with larger sample size, preferably a prospective cohort study should be pursued to confirm our findings and establish any other distinct relationships between the bone remodeling proteins in the OPG/RANKL/RANK axis and bone density in subjects with nephrolithiasis. The other limitation is that non- nephrolithiasis individual were not enrolled as controls.

## Conclusion

The present study showed that high serum fasting OPG levels may be indicative of femoral neck BMD in patients with nephrolithiasis. A deeper knowledge of mechanisms involved in the association between OPG serum levels, bone integrity, and kidney stone disease can provide more robust scientific approaching for future therapeutic interventions.
